# Assessment of a panel of interleukin-8 reporter lung epithelial cell lines to monitor the pro-inflammatory response following zinc oxide nanoparticle exposure under different cell culture conditions

**DOI:** 10.1186/s12989-015-0104-6

**Published:** 2015-09-29

**Authors:** Linda C. Stoehr, Carola Endes, Isabella Radauer-Preiml, Matthew S. P. Boyles, Eudald Casals, Sandor Balog, Markus Pesch, Alke Petri-Fink, Barbara Rothen-Rutishauser, Martin Himly, Martin J. D. Clift, Albert Duschl

**Affiliations:** Department of Molecular Biology, University of Salzburg, Hellbrunnerstrasse 34, 5020 Salzburg, Austria; Grimm Aerosol Technik GmbH & Co. KG, Ainring, Germany; BioNanomaterials, Adolphe Merkle Institute, Université de Fribourg, Fribourg, Switzerland; Institut Català de Nanotecnologia (ICN), Bellaterra, Spain; Soft Matter Scattering, Adolphe Merkle Institute, Université de Fribourg, Fribourg, Switzerland

**Keywords:** A549 cells, Interleukin-8, Air-liquid interface, Submerged cultures, Acute pulmonary (pro-)inflammatory effects

## Abstract

**Background:**

Stably transfected lung epithelial reporter cell lines pose an advantageous alternative to replace complex experimental techniques to monitor the pro-inflammatory response following nanoparticle (NP) exposure. Previously, reporter cell lines have been used under submerged culture conditions, however, their potential usefulness in combination with air-liquid interface (ALI) exposures is currently unknown. Therefore, the aim of the present study was to compare a panel of interleukin-8 promoter (pIL8)-reporter cell lines (*i.e.* green or red fluorescent protein (GFP, RFP), and luciferase (Luc)), originating from A549 lung epithelial type II-like cells cells, following NPs exposure under both submerged and ALI conditions.

**Methods:**

All cell lines were exposed to zinc oxide (ZnO) NPs at 0.6 and 6.2 μg/cm^2^ for 3 and 16 hours under both submerged and ALI conditions. Following physicochemical characterization, the cytotoxic profile of the ZnO-NPs was determined for each exposure scenario. Expression of IL-8 from all cell types was analyzed at the promoter level and compared to the mRNA (qRT-PCR) and protein level (ELISA).

**Results:**

In summary, each reporter cell line detected acute pro-inflammatory effects following ZnO exposure under each condition tested. The pIL8-Luc cell line was the most sensitive in terms of reporter signal strength and onset velocity following TNF-α treatment. Both pIL8-GFP and pIL8-RFP also showed a marked signal induction in response to TNF-α, although only after 16 hrs. In terms of ZnO-NP-induced cytotoxicity pIL8-RFP cells were the most affected, whilst the pIL8-Luc were found the least responsive.

**Conclusions:**

In conclusion, the use of fluorescence-based reporter cell lines can provide a useful tool in screening the pro-inflammatory response following NP exposure in both submerged and ALI cell cultures.

**Electronic supplementary material:**

The online version of this article (doi:10.1186/s12989-015-0104-6) contains supplementary material, which is available to authorized users.

## Background

Analysis of nanoparticle (NP)-induced immune responses *in vitro* often requires elaborate and time-consuming assays such as quantitative reverse transcription polymerase chain reaction (qRT-PCR) and enzyme-linked immunosorbent assay (ELISA). The use of reporter cell lines may circumvent such time-consuming procedures, as the detection signal produced during the activation of intracellular signaling pathways of interest is *via* a concomitant expression of the reporter gene – often encoding either luciferase (Luc) or a fluorescent protein – which can subsequently be quantified using simple light-based detection.

So far, the application of reporter cells has been found to benefit many fields of research, including studies of basic cell mechanisms [1, 2] and cellular stress [3], in understanding molecular mechanisms within disease models [4], for cancer research [5], stem cell research [6], drug development [3], and in the assessment of chemicals [7]. In addition, this technology is often considered to add value when establishing methods for high-throughput screening and expression profiling [3, 8].

In the context of particle toxicology, reporter cell-based assays have proven useful in the assessment of NP-derived immune responses. Several studies have reported the use of luciferase reporter cell lines to assess cellular immune modulation in response to gold (Au), carbon, silver (Ag), silica (SiO_2_) and metal(−oxide) NPs [9–13] and some have validated the observed promoter activity with conventional methods (e.g. qRT-PCR and ELISA), showing good correlation between reporter assays and secreted cytokine analysis [7, 10, 11]. These findings strongly indicate that this technology could be a useful screening method to monitor alterations of the immune status of a cell in response to NP exposure [14]. Fluorescence-based reporter cells have also recently been used to detect additional biochemical endpoints, including oxidative stress and genotoxicity. For example, Fendyur and colleagues assessed the ability for Ag-NPs to induce reactive oxygen species (ROS)-associated DNA damage in NIH-3 T3 cells [15], whilst Karlsson *et al.* [16] investigated the impact of copper (CuO), zinc (ZnO) and nickel oxide (NiO) NP exposure on mouse embryonic stem cells using green fluorescent protein (GFP) to quantify DNA damage and oxidative stress associated with metal oxide-induced cytotoxicity. Furthermore, the adaptability of fluorescence-based reporter cell lines has been highlighted in regards to their culture conditions. As demonstrated by Kohl *et al.* [17], it was possible to culture pIL8-GFP-A549 cells within a novel micro-culture chamber and subsequently deduce the pro-inflammatory responses to Au, Ag, and magnetite NPs with a microscopy-based approach at the single-cell level.

For the majority of NP studies concerning pulmonary health, cells are exposed by directly adding the NP suspension to the cell culture medium covering the cells. This system does not appropriately reflect the *in vivo* situation within lung alveoli, where the alveolar tissue barrier is exposed to air and only covered by a thin liquid lining layer topped with a surfactant film. Furthermore, changes in NP agglomeration, corrosion and dissolution often occur during submerged exposure [18, 19], which in turn makes it difficult to determine and control the delivered dose. This discrepancy between administered and delivered doses in submerged systems has been a subject of many reviews and several dosimetry models based on diffusion and sedimentation have been described [20–22]. In order to circumvent these issues, a number of studies have recently taken to investigate NP-induced pulmonary effects by exposing cells at the ALI to aerosolized NPs, allowing a more realistic interaction between cells and NPs, limiting alterations of the physicochemical properties of NPs, and providing a more accurate dose determination. Various ALI exposure systems have been described, and for a detailed comparison various reviews are available [23, 24].

Due to the above-mentioned flexibility and versatility of reporter cell lines, it is proposed that particularly NP aerosol exposure systems may greatly benefit. As these are historically more complex to conventional submerged systems such an intrinsic complexity could be offset by including a simple, rapid detection of NP aerosol-induced immune responses. This is particularly appropriate since the available literature concerning ALI exposure systems identifies that the main parameters analyzed were pro-inflammatory effects by means of qRT-PCR and ELISA, creating a workload which could be significantly reduced by the use of reporter cell lines. However, reporter cells have not been tested at the ALI so far and their suitability in this set-up remains to be investigated.

In the present study, a suite of reporter cell lines aiming at different applications for pulmonary nanotoxicological assessment were investigated for ZnO-NP-induced pro-inflammatory activation in comparison to non-transfected cells, both under submerged conditions and at the ALI. The (pro-)inflammatory chemokine, interleukin(IL)-8 was chosen as the promoter since it represents an early indicator of the (pro-)inflammatory cascade and has been shown to be up-regulated in response to a number of NPs [25–28], including ZnO [29, 30]. In order to study NP-induced effects upon inhalation, lung epithelial cells are often employed since these cells represent barrier, target and (together with alveolar macrophages) first line of cellular defense against inhaled NPs [31]. Therefore, three reporter cell lines (GFP; red fluorescent protein, RFP; Luc) derived from the lung alveolar epithelial cell line A549 were used for the intended profiling of ZnO-NP-induced IL-8 induction. Determined parameters included: (i) differences in the promoter activity of the reporter cells, (ii) how reporter cells compared to non-transfected counterparts (A549), (iii) whether reporter activity could be validated with conventional methods such as qRT-PCR and ELISA, and (iv) which reporter cells are most appropriate for use in a submerged and ALI exposure system, using the previously described air-liquid interface cell exposure system (ALICE) [29].

## Results

### Particle characterization

Size distribution and aggregation of ZnO-NPs upon dispersion in different media and after nebulization are summarized in Table 1. Particle sizes determined from TEM images of NPs in H_2_O (Additional file 1: Figure S1) were approximately 30–40 nm, correlating well with the manufacturer’s information (35 nm mean size, <100 nm), and only a low degree of agglomeration was observed.

**Table 1:** Particle characteristics

	Size DLS [nm]	Size TEM [nm]	Z-potential [mV]
in H_2_O	138 ± 62	30–40	26.5 ± 0.3
ALI^a^	67 ± 25	partially agglomerated	n.d.
in CCM	1000 ± 400	100–2000	n.d.

The ZnO-NP suspension (50 % in H_2_O, average mean size 35 nm, < 100 nm (DLS)) was obtained from Sigma and characterized in H_2_O as well as in cell culture medium (CCM; RPMI + 10 % FCS). Zeta potentials were not determined (n.d.) in PBS or CCM

^a^DLS: prior to nebulization in NaCl solution; TEM: after nebulization

Careful visual inspection confirmed that ZnO-NPs agglomerated quickly in CCM, and the rapid formation of a sediment layer on the bottom of the vial was observed (data not shown). Indeed, a representative TEM micrograph (Additional file 1: Figure S1) shows clear evidence of highly heterogeneous agglomerates of various sizes. DLS analysis further estimated a typical size of approximately 1000 nm with considerable polydispersity.

The zeta potential of ZnO-NPs in H_2_O yielded an average surface charge of +26.5 ± 0.3 mV. Furthermore, TEM showed a homogeneous distribution of nebulized NPs as well as few agglomerates (Additional file 1: Figure S2).

### Dissolution of ZnO-NPs in aqueous media

Dissolution of ZnO-NPs in CCM was investigated *via* ICP-MS (Additional file 1: Table S1). Depending on initial concentration, the ZnO-NPs dissolved in CCM, whereby 90 % dissolution was reached for 1.1 μg/ml vs. 55 % for 21.2 μg/ml already after 3 hours.

### Determination of concentrations for submerged exposure experiments

The concentrations used for submerged exposures were chosen to match the deposited concentrations measured during ALI exposure (low_ZnO_ = 0.6, high_ZnO_ = 6.2 μg/cm^2^) and administered at doses depending on the well-format used for the assays (details are given in Table 2). Due to the agglomeration of the ZnO-NPs in CCM, as observed by both TEM and DLS, the time-dependent deposition of mass is expected to be dominated by gravitational settling [20–22, 32, 33], which led to a practically complete mass deposition within 3 hours. A representative settling velocity is reported in the Additional file 1: Supplementary Material.

**Table 2:** Nanoparticle doses

ZnO-NPs	Assays	[μg/ml]		[μg/well]		[μg/cm^2^]	
concentration		low_ZnO_	high_ZnO_	low_ZnO_	high_ZnO_	low_ZnO_	high_ZnO_
ALI^a^	all					0.62	6.23
96-well submerged	Cytotoxicity & reporter assays	2.1	21.2	0.21	2.12	0.62	6.24
24-well submerged	qRT-PCR & ELISA	1.2	11.8	1.2	11.8	0.63	6.21
6-well submerged	CLSM		13.1		26.2		6.23

^a^as determined by QCM. Particle concentrations for submerged experiments were calculated based on the complete settling of NPs within the investigated incubation times. Growth areas were 0.33 cm^2^ for 96-well plates, 1.9 cm^2^ for 24-well plates and 4.2 cm^2^ for 6-well insert. Administered volumes were 0.1 ml, 1 ml and 2 ml, respectively

### Cytotoxic effects of ZnO-NPs

Cytotoxicity caused by ZnO-NPs under submerged and ALI exposure conditions was investigated *via* the lactate dehydrogenase (LDH) release assay.

**Fig. 1:**
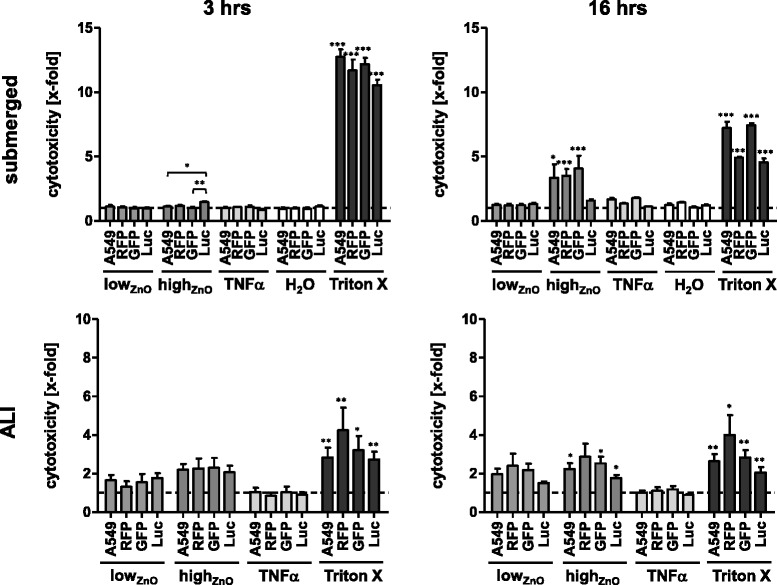
Evaluating cytotoxicity of ZnO nanoparticles under submerged and ALI conditions. A. Cytotoxicity as determined by LDH release assay. Data are presented as mean x-fold increase over untreated control (submerged) or NaCl-nebulized control (ALI) (dashed line). Error bars indicate the SEM of at least three independent experiments. A one-way analysis of variance (ANOVA) with a subsequent Tukey’s Multiple Comparison test was performed. Values were considered significantly different compared to the unexposed (submerged) or NaCl-nebulized (ALI) control or as indicated with *p* < 0.05 (*), *p* < 0.001 (**) and *p* < 0.0001 (***)

*Submerged* – None of the cell lines showed increased cytotoxicity after 3 hours following ZnO-NP treatment (Fig. 1). After 16 hours, RFP, GFP, as well as non-transfected cells (A549) showed a significant increase in LDH release (RFP: 3.5 ± 0.5-fold; A549: 3.3 ± 1.1-fold; GFP: 4.1 ± 1.0-fold) in response to high_ZnO_, whereas Luc showed only a slight increase (1.6 ± 0.1-fold) that was not found to be statistically significant. RFP and Luc had slightly higher baseline levels of LDH release compared to A549 and GFP respectively. Treatment with the positive pro-inflammatory control, TNF-α, for 16 hours induced a slight (~1.5-fold) but insignificant increase in LDH release in both A549 and GFP.

*ALI* – Upon ZnO exposure, LDH release increased in all cell lines for both particle concentrations, although these changes were not significant after 3 hours. After 16 hours, significant LDH release levels were found following high_ZnO_ exposure to Luc (1.8 ± 0.2-fold), A549 (2.2 ± 0.3-fold) and GFP (2.5 ± 0.3-fold) cells, although non-significant in RFP (2.9 ± 0.7-fold).

### Acute immune effects caused by ZnO-NPs

#### Reporter gene assays

**Fig. 2:**
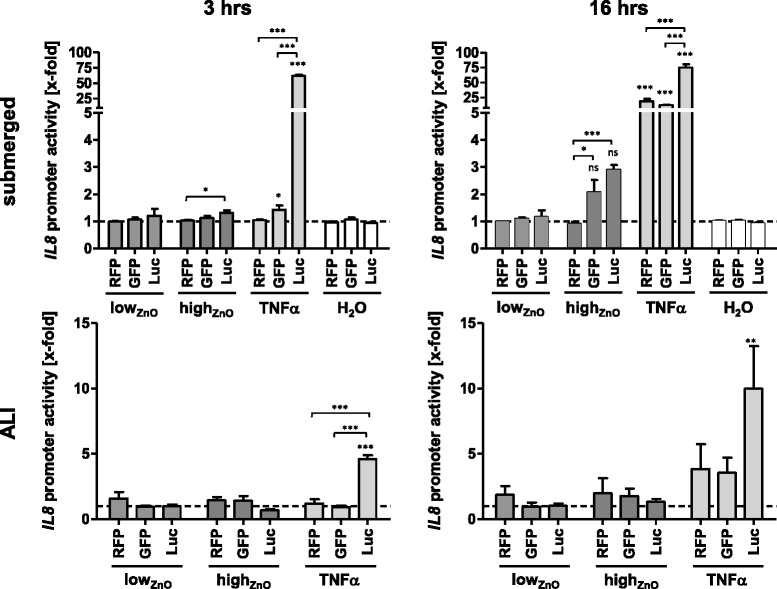
Pro-inflammatory response upon exposure to ZnO-NPs monitored by p*IL8* A549 reporter cell lines under submerged and ALI conditions. Data are presented as mean x-fold increase over untreated control (submerged) or NaCl-nebulized control (ALI) (dashed line). Error bars indicate the SEM of at least three independent experiments. A one-way analysis of variance (ANOVA) with a subsequent Tukey’s Multiple Comparison test was performed. Values were considered significantly different compared to the unexposed (submerged) or NaCl-nebulized (ALI) control or as indicated with *p* < 0.05 (*), *p* < 0.001 (**) and *p* < 0.0001 (***); Ns = not significant

*Submerged* – None of the cells showed a significant increase in *IL8* promoter activity in response to ZnO-NPs after 3 hours (Fig. 2). In response to TNF-α, a strong signal could only be detected for Luc (62.1 ± 1.8-fold) at this early time point, although a slight, statistically significant, increase (1.4 ± 0.2-fold) could be detected for GFP as well. After 16 hours, an increase of *IL8* promoter activity could be observed for both Luc and GFP exposed to high_ZnO_ (Luc: 2.9 ± 0.2-fold; GFP: 2.1 ± 0.4-fold), which was significant only for GFP. RFP did not show any effect, which could be explained by the observed high cytotoxicity of high_ZnO_ at this exposure time. Concomitant with the observed differences in ZnO-NP susceptibility, the responses to TNF-α was different across the cell lines. While *IL8* promoter activity was 75.2 ± 5.5-fold increased for Luc, RFP and GFP showed increases of 19.1 ± 3.8-fold and 12.6 ± 0.7-fold, respectively.

*ALI* – A similar trend in the onset of *IL8* promoter activity in TNF-α-induced cells could be found after 3 hours as for the submerged exposures. Only Luc showed a significant 4.6 ± 0.3-fold increase, which was much lower than in the submerged exposure. While this might partly be caused by the different exposure method, it could also be further reduced by the indirect contact between cells and TNF-α due to the membrane of the insert. None of the cells showed any *IL8* promoter induction upon exposure to ZnO-NPs at this early time point. Furthermore, after 16 hours no response in the ZnO-exposed cells could be observed, which again might have been due to the observed cytotoxicity. Again, Luc showed the highest increase in *IL8* promoter activity upon treatment with TNF-α (10 ± 3.3-fold), whereas RFP and GFP reached only approximately 4-fold increases (3.8 ± 1.9-fold; 3.6 ± 1.2-fold; both p > 0.05).

### IL8 mRNA expression

**Fig. 3:**
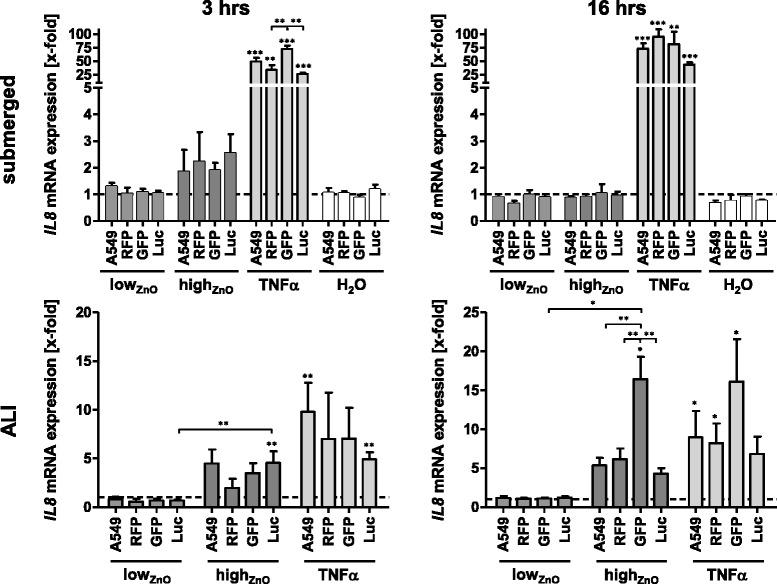
*IL8* gene expression upon exposure to ZnO-NPs under submerged and ALI conditions monitored by qRT-PCR. Data are presented as mean x-fold increase over untreated control (submerged) or NaCl-nebulized control (ALI) (dashed line). Error bars indicate the SEM of at least three independent experiments. A one-way analysis of variance (ANOVA) with a subsequent Tukey’s Multiple Comparison test was performed. Values were considered significantly different compared to the unexposed (submerged) or NaCl-nebulized (ALI) control or as indicated with *p* < 0.05 (*), *p* < 0.001 (**) and *p* < 0.0001 (***)

*Submerged* – High_ZnO_ induced an approximately 2-fold increase of *IL8* mRNA expression in all cell lines after 3 hours, compared to untreated controls, but these were not statistically significant (Fig. 3). After 16 hours, these effects were reduced, closer to baseline, indicating a transient response which might be related to the fact that mRNA can be readily degraded, if a stimulus is not persistent enough [34]. Neither the H_2_O solvent control nor low_ZnO_ induced changes in *IL8* mRNA expression at either time point. No significant differences between the individual cell lines could be detected for the respective treatments.

*ALI* – Upon exposure to high_ZnO_, *IL8* mRNA expression was increased in all cell lines after 3 hours, which was only significant for Luc (4.6 ± 1.2-fold). Expression levels upon stimulation with TNF-α also varied between the cell lines, ranging from 9.8 ± 3.0-fold (A549) to 4.9 ± 0.7-fold (Luc) compared to NaCl-nebulized controls, with the fluorescent cells in-between (RFP: 7.0 ± 4.8-fold; GFP: 7.0 ± 3.2-fold; p > 0.05). After 16 hours, increased *IL8* mRNA expression levels were detected in all cell lines upon exposure to high_ZnO_. While RFP, Luc and A549 reacted quite similarly (4-6-fold increases; *p* > 0.05), a much higher induction (16.4 ± 2.9-fold) was observed in GFP, which was also statistically significant when compared to the other cell lines at this treatment. None of the cell lines were affected by low_ZnO_. Treatment with TNF-α for 16 hours resulted in increased *IL8* mRNA levels for all cell lines (GFP: 16.1 ± 5.5-fold, A549: 9.0 ± 3.4-fold, RFP: 8.2 ± 2.5-fold, all *p* < 0.05; Luc: 6.8 ± 2.2-fold, *p* > 0.05).

#### IL-8 protein release

**Fig. 4:**
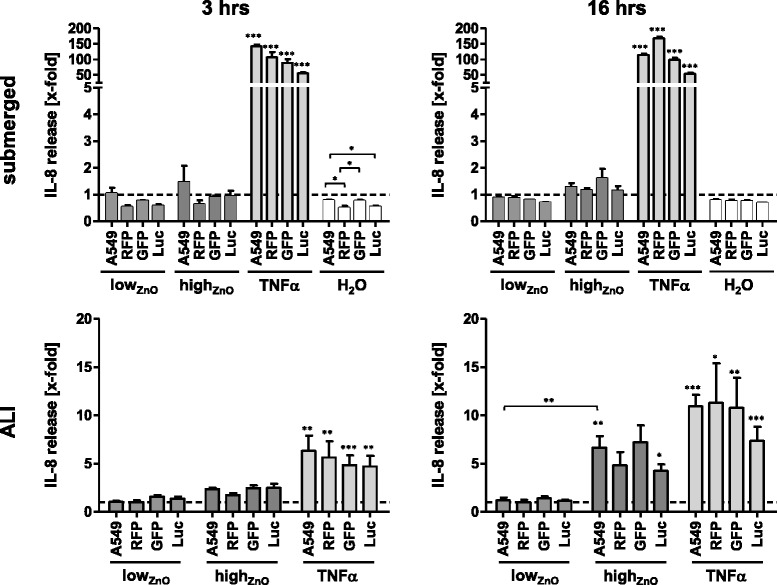
Secretion of IL-8 upon exposure to ZnO-NPs under submerged and ALI conditions monitored by ELISA. Data are presented as mean x-fold increase over untreated control (submerged) or NaCl-nebulized control (ALI) (dashed line). Error bars indicate the SEM of at least three independent experiments. A one-way analysis of variance (ANOVA) with a subsequent Tukey’s Multiple Comparison test was performed. Values were considered significantly different compared to the unexposed (submerged) or NaCl-nebulized (ALI) control or as indicated with p < 0.05 (*), *p* < 0.001 (**) and *p* < 0.0001 (***)

*Submerged* – No significant effects on IL-8 release could be seen for any of the cell lines after treatment with both ZnO-NP doses at the tested time points (Fig. 4). The solvent control (H_2_O) seemed to reduce IL-8 release. While this was not significant in comparison to CCM controls, significant differences between the cell lines were detected after 3 hours.

*ALI* – High_ZnO_ induced approximately 2.5-fold increase in IL-8 release in all cell lines except for RFP (1.7-fold) after 3 hours. After 16 hours, this effect was even more prominent, showing a 6–7-fold increase for GFP and A549, as well as 4–5-fold increases for RFP and Luc. However, these increases were only significant for A549 (6.7 ± 1.2-fold) and Luc (4.3 ± 0.7-fold) in comparison to controls. Low_ZnO_ did not affect the cells during the observed periods of time. It is noteworthy that there were no differences between cells exposed to aerosolized NaCl (control) in the exposure chamber and ones kept in the incubator (not shown). IL-8 release upon stimulation with TNF-α was significantly increased after both time points and at similar levels in all cell lines (5–6-fold, 3 hours; 7–11-fold, 16 hours).

## Discussion

The increasing number of engineered NPs calls for faster and easier screening methods for biosafety assessment, especially in the field of inhalation toxicology. Reporter cell lines facilitate easy light emission-based detection of immune responses, and could significantly decrease the workload given by complex biochemical assays. In addition, they offer options for single-cell studies, and they are also attractive for complex culture systems since the signal can be unambiguously traced to a specific cell type. As these cell lines have not been tested at the ALI yet, the purpose of this study was to compare the performance of a panel of A549-derived reporter epithelial cell lines carrying different reporter genes for *IL8* promoter induction upon exposure to NPs under submerged conditions and at the ALI. A secondary objective was to evaluate the applicability of the reporter cell lines in place of more elaborate techniques (qRT-PCR, ELISA) in terms of sensitivity, signal onset velocity, and robustness.

### Rationale for IL8 as pro-inflammatory marker in pulmonary nanotoxicology

Using the established air-liquid interface cell exposure system (ALICE), which has been employed previously to expose lung epithelial cells to a variety of NPs such as ZnO-, Au-, and Ag-NPs [29, 35, 36], A549 lung cells were exposed to ZnO-NPs at the ALI. ZnO-NPs were chosen because they have previously been shown to induce IL-8 expression in human alveolar epithelial cells (A549) in several *in vitro* studies, under both submerged and ALI conditions [29, 30, 37]. Lenz *et al.* investigated the effects of ZnO-NPs with a primary diameter of 24–71 nm under submerged conditions as well as at the ALI using the ALICE [29]. When comparing both exposure scenarios, they found significant differences in *IL8* mRNA expression for the highest dose (8.5 μg/cm^2^ ALICE; 5.0 μg/cm^2^ submerged) after 3 hours incubation, whereby a higher response was observed under submerged conditions. The same type of ZnO-NPs was used by Lenz *et al*. in another study using a different ALI exposure system, previously described by Bitterle *et al*. [38]. Here, however, it was found that transcription of IL-8 and other pro-inflammatory markers (IL-6, GM-CSF) were up-regulated to higher levels upon ZnO-NPs exposure (0.7 and 2.2 μg/cm^2^) under ALI conditions than under submerged conditions [30]. Hsiao *et al.* [37] investigated release of IL-8 after treatment with ZnO-NPs (50–70 nm primary size) for 24 hours and observed significant increases starting from 20 μg/ml. Similarly, Yan *et al.* [39] found significantly increased *IL8* mRNA and protein expression levels in BEAS-2B cells after submerged exposure to ZnO-NPs (30 nm) for 2–8 hours at concentrations starting from 1 μg/cm^2^, which further increased in a dose-dependent manner. Consistent with these previous findings, ZnO-NPs induced IL-8 expression in the present study, and the responses differed by the exposure method used as well as the parameter analyzed.

### Challenges for comparing different exposure setups

Additional file 1: Table S2 gives an overview on the cellular responses and determined endpoints for the two experimental setups that were investigated in the present study. Besides the issues of agglomeration and uncertain dosing in the submerged exposure system, which was addressed *via* calculation of the sedimentation rates and adjusting the administered doses appropriately, the release of Zn^2+^ ions from the NPs might have influenced the observed effects. Dissolution of ZnO-NPs in aqueous media has previously been reported and the toxicity of these NPs has been described to be partially, if not largely, due to the release of toxic Zn^2+^ ions [40–42]. In line with these studies, a significant release of Zn^2+^ from the particles was detected upon incubation in CCM, indicating that the responses might have been caused by the released ions. This was further confirmed by ion control experiments, which showed similar *IL8* promoter induction in cells treated with CCM pre-incubated with ZnO-NPs and cells treated with the corresponding amounts of Zn^2+^ from zinc sulphate (Additional file 1: Figure S5). Although it cannot be ruled out that some NPs remained in the supernatant after centrifugation, possibly contributing a particle effect to the observed responses, these findings suggest that the ZnO-NPs effects on IL-8 expression were primarily caused by ions, which is, as the agglomeration mentioned above, an issue to be considered for submerged exposure. The dissolution rate of ZnO-NPs at the ALI is considered to be much lower, as well as slower, thus induce time-delayed responses. However, release of ions does not seem to be the only way of ZnO-NPs to display toxicity, and previous findings have suggested distinct mechanisms of ZnO toxicity upon exposure at the ALI and under submerged conditions [43].

### Comparison of different reporters

This study compared for the first time three different reporter systems (Luc, GFP, RFP) under control of the *IL8* promoter in two exposure systems. Upon treatment with TNF-α, the pIL8-Luc cell line was found to be the most sensitive showing the earliest onset of expression and highest signal strength under both submerged and ALI conditions. This early onset is consistent with time-response curves in the validation study of the pIL8-Luc cell line, which showed first significant increases after similar exposure times (4 hours) [7], and is regarded a hallmark of the luciferase reporter. Considering the enzymatic nature of the assay and very low background noise (cells usually do not express luciferase), luciferase can already be detected at low amounts, whereas green fluorescent proteins typically need to be present at much higher concentrations in order to be detectable over the background noise caused by cellular autofluorescence [44, 45]. Using RFP instead of GFP has the advantage of less background fluorescence, which was confirmed in flow cytometry experiments (see Supplementary Material). This lower autofluorescence was less obvious in the microplate reader format, most likely due to analyzing the entire cell population, whereas the flow cytometer disregards non-viable cells. Although particularly fast-maturing and bright variants of fluorescent proteins (TurboGFP/RFP) were used, it seems likely that the accumulation time still exceeded the time needed to produce detectable amounts of luciferase since the fluorescent proteins lack the multiplication effect of Luc that is active as an enzyme. Consistent with this, assessment of fluorescent reporter expression is typically performed after exposure for 12 hours and longer [15, 17]. Furthermore, the vector for luciferase differed from the ones used for the fluorescent proteins, which can also influence responsiveness, e.g. due to better transfection efficiency or slight differences in promoter region sequences altering the number of copies of the expression vector integrated into the genomic DNA [7].

### Suitability of reporter cell lines as valid alternative readouts in nanotoxicology

As expected, there were some differences between IL-8 expression on promoter, mRNA and protein levels. Typically, mRNA production precedes protein synthesis, and both biochemical processes can be independently regulated, e.g. by mRNA degradation or post-transcriptional processing. Hence, following a chronological order, mRNA is expected to show up first, followed by the (intracellularly expressed) reporter and finally the newly synthesized and secreted cytokine. This exact pattern was observed for mRNA and reporter protein under submerged conditions in our study, whereas no cytokine secretion could be detected throughout the investigated incubation times, but most likely would have shown up at later time points. Previous studies have used incubation times of 24 hours or longer to assess protein secretion *via* ELISA [10, 11, 14]. This was, however, not suitable for the large comparative study intended here, whilst 3 and 16 hours were selected before-hand as a compromise to meet at best the requirements for all selected endpoints, exposure systems, and reporters. Of note, mRNA and reporter can be transiently expressed, whereas ELISA measures secreted protein which has accumulated over the whole exposure time [10]. Thus, a good agreement between mRNA expression and reporter assays may be preferred when the dynamics of NP-induced effects are investigated.

Additional studies using reporter constructs, such as for markers of DNA damage and oxidative stress [15, 16], were mostly validated indirectly, e.g. by comet assay or ROS measurements, rather than a specific assessment of the actual gene of interest by several endpoints. As this study included three reporters (RFP, GFP, Luc), two exposure systems (ALI, submerged), two exposure times (3, 16 hours), two concentrations resulting in various delivered doses (as discussed above), and three endpoints (mRNA production, reporter expression, IL-8 secretion), this study presents a comprehensive survey of relevant parameters.

## Conclusions

This study gives an overview on influencing factors, experimental challenges, and considerations for selecting the most suitable reporter system in pulmonary nanotoxicology. By using optimized exposure times, reporter assays based on stably transfected lung epithelial type II-like cells provide a clear advantage over time-consuming procedures such as qRT-PCR and ELISA. They further enable detection of transient responses over a wider time frame, whilst reducing workload and instrumentation needs. Finally, reporter cell lines allow for a rapid toxicity screening of NPs, permit live monitoring of cellular stress during on-site safety assessment, and are suitable to be applied at ALI-based exposure conditions.

## Methods

### Cell lines

Three stably transfected cell lines derived from the human lung alveolar adenocarcinoma cell line A549 (American Type Culture Collection; Manassas, USA) containing different reporter genes for Interleukin (IL)-8 expression (Luc, RFP, GFP), as well as non-transfected A549 cells, were used. The generation of the pIL8-Luc-A549 and pIL8-GFP-A549 cell lines has previously been described [7, 17] and details on the pIL8-RFP-A549 cell line can be found in the Supplementary Material. All cell lines were cultured at 37 °C in a humidified incubator with 5 % CO_2_.

### Nanoparticles

#### Characterization

ZnO-NPs (50 % in H_2_O, ~35 nm diameter, <100 nm; Sigma-Aldrich, St. Louis, MO, USA) were characterized as obtained (in H_2_O), in cell culture medium (CCM; for submerged experiments) and after nebulization in the ALICE (for ALI exposure). Particle size was assessed using transmission electron microscopy (TEM) and dynamic light scattering (DLS), whilst surface charge was determined by measuring their zeta-potential.

Size distribution and zeta-potential were measured using a 3D LS Spectrometer (LS Instruments, Fribourg, Switzerland) and a Malvern Zetasizer Nano ZS (Malvern Instruments Ltd., Worcestershire, UK). Size measurements were performed upon freshly diluted particles in H_2_O or medium and their zeta-potential was measured in H_2_O.

Furthermore, TEM analysis for particles suspended in H_2_O and in medium was performed using a ZeissLEO 912AB (Zeiss, Oberkochen, Germany) at 80 kV.

To investigate agglomeration state and size of nanoparticles after nebulization in the ALI experiments, protein pre-coated TEM grids were placed into the ALICE and the particle suspension was nebulized upon them. The grids were then analyzed using a Hitachi H-7100 (Hitachi, Tokyo, Japan) at 75 kV.

### Ion release from ZnO-NPs under submerged conditions

The particles were incubated in cell culture medium at the appropriate concentrations and incubated at 37 °C/5 % CO_2_ for 3 and 16 hours. Afterwards, the samples were centrifuged at 18.000x*g*, 4 °C for 15 minutes, the particle pellet was discarded and the supernatants were stored at −80 °C. Zinc ion content of the supernatants was assessed by LEITAT Technological Center (Barcelona, Spain) using inductively coupled plasma mass spectrometry (ICP-MS). Analysis was performed using an Agilent 7500cx ICP-MS (Agilent Technologies, Santa Clara, CA, USA) with a detection limit of 0.02386 ppb.

### Cell culture media

*Submerged* – A549 cells were cultured, seeded and exposed in RPMI 1640 medium without L-glutamine (Sigma-Aldrich), supplemented with 10 % fetal calf serum (FCS; PAA, Pasching, Austria), 100 U/ml Penicillin, 100 μg/ml Streptomycin, 2 mM L-glutamine (all Sigma-Aldrich). All transfected cell lines were cultured in A549 medium containing 500 μg/ml gentamycin (G-418 sulphate, Sigma-Aldrich) as selection antibiotic. For exposure of pIL8-RFP-A549 and pIL8-GFP-A549, RPMI medium without phenol red (Gibco®, Life Technologies, Carlsbad, CA, USA) supplemented accordingly was used in order to avoid interference of the indicator dye with the readout.

*ALI* – The cell lines were cultured and exposed in medium supplemented as described above, but RPMI 1640 supplemented with HEPES and without L-glutamine (Gibco® Life Technologies) was used. Penicillin, Streptomycin, L-glutamine and RPMI 1640 without phenol red were purchased from Life Technologies as well. FCS and G-418 was purchased from PAA and Sigma-Aldrich, respectively.

### Preparation of cells for exposure

*Submerged* – Semi-confluent cell layers were rinsed with 1× PBS (Sigma-Aldrich), trypsinized (0.05 % Trypsin-EDTA, GE Healthcare Life Sciences, Little Chalfont, UK), diluted in the appropriate media and seeded at a density of 2.1 × 10^4^ cells/cm^2^ into 24- or 96-well plates, depending on the endpoint assessed. The cells were then left to grow to confluence for 6 days and exposed to NPs in suspension added to the CCM.

For cytotoxicity and reporter gene assays, A549 and pIL8-Luc A549 cells were seeded into 96-well flat-bottom cell culture plates (Costar®, Corning Incorporated, Corning, NY, USA) whereas the fluorescent cell lines were seeded into black 96-well flat-bottom cell culture plates with transparent bottom (μClear®, Greiner Bio-One, Kremsmünster, Austria), using 100 μl of a 7 × 10^4^ cells/ml cell suspension. For flow cytometry, ELISA and PCR analyses, cells were seeded into 24-well flat-bottom cell culture plates (Costar®, Corning Incorporated), using 1 ml of a 3.9 × 10^4^ cells/ml cell suspension.

*ALI* – Cells were rinsed with 1× PBS, detached by trypsinization (0.05 % Trypsin-EDTA, both Gibco® Life Technologies) and 2 ml of a 5 × 10^4^ cells/ml cell suspension were seeded into 6-well Falcon® cell culture inserts (transparent PET membrane, 4.2 cm^2^ growth area, 3 μm pore size, BD Biosciences, Franklin Lakes, NJ, USA) held in 6-well tissue culture plates (Falcon®, BD Biosciences), yielding a density of 2.4 × 10^4^ cells/cm^2^. After an initial growth phase under submerged conditions for five days, the cells were transferred to the ALI by removing the medium in the upper compartment and exchanging the medium in the lower compartment. The next day, immediately prior to exposure in the ALICE, the medium in the lower compartment was replaced with fresh medium.

### Exposure to ZnO-NPs

*Submerged* – In order to match the deposited masses during ALI exposure (monitored using an integrated quartz crystal microbalance (QCM)), the low_ZnO_ and high_ZnO_ doses for submerged exposures were adapted to the well size formats and volumes used as described in Table 1). At the day of exposure, the medium was replaced and after a short recovery period of 15 min, appropriate volumes of freshly prepared 10× particle dilutions in LAL reagent water (CAPE COD Incorporated, East Falmouth, MA, USA) were added in a 1:10 ratio to the cell culture media. The final concentrations are shown in Table 1. The cells were left to incubate in a humidified incubator for 3 and 16 hours at 37 °C/5 % CO_2_. As controls, cells were treated with medium only (negative control), H_2_O (solvent control) or 20 ng/ml recombinant human TNF-α (ImmunoTools, Friesoythe, Germany) to induce an IL-8 response. For cell viability and cytotoxicity assays, 0.1 % Triton X-100 (Bio-Rad Laboratories, Hercules, CA, USA) was used as an additional control to lyze all cells.

*ALI* – The cell cultures were exposed to a low_ZnO_ and high_ZnO_ concentration of nebulized ZnO-NPs in the ALICE as described in the Supplementary Material, and finally placed in a humidified incubator at 37 °C/5 % CO_2_ for additional incubation periods of 3 and 16 hours at the ALI. To confirm any particle-associated effects, control cultures were exposed to nebulized NaCl solution only (negative control). NaCl-only exposed cells stimulated with 20 ng/ml rhTNF-α (Miltenyi Biotec, Bergisch Gladbach, Germany; ImmunoTools) added to the medium beneath the cells served as the positive control for IL-8 induction, whilst Triton X-100 (0.2 %, Merck Millipore, Billerica, Massachusetts, USA) added to the medium beneath the NaCl-only exposed cells was used as the positive control for the cytotoxicity assay.

### Analysis of biological endpoints

Cytotoxicity, *IL8* promoter activity, mRNA expression, and IL-8 release were assessed using commercially available assays according to the manufacturer’s instructions and are described in the Supplementary Material.

### Statistical analysis

All data is presented as the mean ± standard error of the mean (SEM) of at least three independent experiments. All data was analyzed using Microsoft® Office Excel 2007 and imported into GraphPad Prism 5 for plotting and statistical evaluation. Statistical analysis was performed using a one-way analysis of variance (one-way ANOVA) with *post-hoc* testing by Tukey’s Multiple Comparison test. An alpha-value <0.05 was regarded as statistically significant, with *p* < 0.001 = ***, *p* < 0.01 = **, and *p* < 0.05 = *. FACS data was analyzed using FACSDiva v6.1.2 (BD Biosciences).

## Additional file

Additional file 1: **Supplementary Material - Stoehr et al. PFT 2015.** (PDF 838 kb)
