# Supplementary material for: Assessment of a panel of interleukin-8 reporter lung epithelial cell lines to monitor the pro-inflammatory response following zinc oxide nanoparticle exposure under different cell culture conditions
Source: Part Fibre Toxicol. 2015 Sep 29;12:29. doi: 10.1186/s12989-015-0104-6 (PMC4587722; doi:10.1186/s12989-015-0104-6)
Supplement: Supplementary file 1 — Supplementary Material - Stoehr et al. PFT 2015. (PDF 838 kb) [file 12989_2015_104_MOESM1_ESM.pdf]

Supplementary Material

Assessment of a panel of interleukin-8 reporter lung epithelial cell lines to monitor the pro-inflammatory response following zinc oxide nanoparticle exposure under different cell culture conditions

Linda C. Stoehr, Carola Endes, Isabella Radauer-Preiml, Matthew S.P. Boyles, Eudald Casals, Sandor Balog, Markus Pesch, Alke Petri-Fink, Barbara Rothen-Rutishauser, Martin Himly, Martin J.D. Clift, and Albert Duschl

Methods in more detail

Generation of pIL8-RFP-A549 cell line

The pIL8-RFP-A549 cell line was generated by cloning the *IL8* promoter sequence from the pIL8-Luc vector into a promoter-less expression vector containing both the RFP sequence and a gentamycin resistance gene (pTurboRFP-PRL; Evrogen, Moscow, Russia). A549 cells were then transfected using Lipofectamine® 2000 (Life Technologies, Carlsbad, CA, USA) according to the manufacturer's instructions. RFP-positive clones were singled out and expanded in selection medium containing G-418.

Dosimetry calculations for submerged exposure

In order to match the deposited masses during ALI exposure, the low_{ZnO} and $high_{ZnO}$ doses for submerged exposures were adapted to the well size formats and volumes used (**Table 2**). As the DLS measurements revealed a fast agglomeration of the NPs in medium, mass deposition was expected to be driven mainly by gravitational settling. A modification of Stoke's Law was used to calculate the settling velocity of the particle agglomerates (polydisperse fractal clusters) based on the diameter of approximately 1000 nm determined by DLS resulting in approximately $5x10^{-7}$ m/s [1, 2].

With the administered volumes of 0.1 ml for 96-well (0.33 cm^2 growth area, resulting in 0.3 cm height of the liquid) and 1 ml for 24-well plates (1.9 cm^2, resulting in 0.52 cm height of the liquid), settling times being well within the observed time frames were estimated. Therefore, the delivered dose after 3 hours was expected to be equal with the one after 16 hours and no adjustments needed to be made in the administered doses for the two exposure times. Based on the growth areas, the administered doses were calculated to be 2.1 and 21.2 µg/ml for the 96-well format (0.33 cm^2 growth area) and 1.2 and 11.8 µg/ml for the 24-well format (1.9 cm^2), resulting in approximately 0.62 and 6.2 µg/cm^2. A comparison of the doses can be found in **Table S3**.

Submerged exposures

Cytotoxicity assay

Cytotoxicity was assessed by measuring LDH release from damaged cells using the CytoTox96® Non-Radioactive Cytotoxicity Assay (Promega, Fitchburg, WI, USA). A volume of 30 µl of supernatant was added into a transparent 96-well plate (Nunc Maxisorp®, eBioscience, San Diego, USA) and 30 µl of substrate was added. After incubation for 30 min in the dark, the reaction was stopped by adding 30 µl stop solution and absorbance was recorded at 490 nm in a microplate reader (Infinite® 200 PRO, Tecan, Grödig, Austria).

Confocal laser scanning microscopy

To complement the cytotoxicity data, the cellular morphology was investigated by confocal laser scanning microscopy (CLSM). Details are given in the 'ALI exposure' section below.

IL8 reporter gene assays

After removing the supernatants for LDH analysis, cells were prepared for gene reporter assays.

To analyze *IL8* promoter activity of the fluorescent reporter cell lines pIL8-RFP A549 and pIL8-GFP A549, the cells were washed once with 100 µl PBS (Sigma-Aldrich) and another 100 µl of PBS was

added. Fluorescence was recorded at $540_{Ex}/574_{Em}$ nm for RFP and $478_{Ex}/520_{Em}$ nm for GFP using a microplate reader (Infinite® 200 PRO, Tecan). As interference control, PBS from wells incubated with nanoparticles in medium without cells was used and subtracted as background fluorescence.

To analyze *IL8* promoter activity of the luminescent reporter cell line pIL8-Luc A549, the cells were washed once with 100 µl PBS (Sigma-Aldrich) and lyzed using 50 µl Lysis Buffer (90.8 mM K_2HPO_4, 9.2 mM KH_2PO_4 and 0.5% Triton X-100, in Millipore H_2O, adjusted to pH 7.8 with KOH and supplemented with freshly added 1 mM DTT). After incubation at 37°C/5% CO_2 for 15 minutes, the assay was performed as previously described [3].

Flow cytometry analysis

To complement the plate reader measurements, which only give a read-out of the entirety of cells in a well, the fluorescence intensity of single cells was analyzed by flow cytometry. For this, pIL8-RFP or pIL8-GFP cells were seeded into 24-well plates and exposed as described above. After 3 or 16 hours of incubation, cells were washed with 1x PBS (1 ml/well) and detached by trypsinization (100 µl/well for 5 min). A volume of 400 µl supplemented medium without phenol red was added and the cells were transferred into 5 ml round-bottom polystyrene tubes (BD Falcon™, BD Biosciences, Franklin Lakes, NJ, USA). Fluorescence intensity and number of GFP- or RFP-positive cells were recorded using a BD FACS Canto III (BD Biosciences). For each treatment 10'000 cells were analyzed (stopping gate), with the gate limits set to transfected non-fluorescent cells (pIL8-Luc A549).

IL8 mRNA expression

Gene expression of interleukin-8 at the mRNA level was analyzed using quantitative reverse transcription polymerase chain reaction (qRT-PCR). All cell lines were seeded and incubated with nanoparticles for 3 and 16 hours as described above. After removing the supernatants, the cells were rinsed once with 1x PBS (1 ml/well) and 500 µl of TRI Reagent (Sigma-Aldrich) were added to each well. After a short incubation period on ice, the samples were stored at -80°C. Total RNA was

isolated by phenol/chloroform extraction and precipitated using isopropanol. After washing with ethanol, the pellet was dissolved in RNase-free H_2O and stored at -20°C until use. Single-stranded cDNA was synthesized from 4 µg oligo dT-primed RNA by reverse transcription using RevertAid H Minus Reverse Transcriptase (Fisher Scientific, Waltham, MA, USA) in a thermal cycler (Mastercycler®, Eppendorf, Hamburg, Germany). Afterwards, the appropriate specific primer mixes and iQ™ SYBR® Green Supermix (Bio-Rad Laboratories) were mixed with the template cDNA and quantitative real-time PCR was performed in a Rotor-Gene RG-3000 (Corbett Life Science/Qiagen, Hilden, Germany)

The housekeeping gene large ribosomal protein P0 (*RPLP0*) was used to normalize the values to an internal control. Primers used were *IL8*: commercially available IL8 RT² qPCR Primer Assay (Qiagen, Hilden, Germany), and *RPLP0*: fwd 5'GGCACCATTGAAATCCTGAGTGATGTG3' and rev 5'TTGCGGACACCCTCCAGGAAG3' (synthesized by Sigma-Aldrich).

IL-8 protein release

The amount of the pro-inflammatory IL-8 protein released by the cells after exposure to ZnO nanoparticles was quantified *via* ELISA. All cell lines were seeded and incubated with nanoparticles for 3 and 16 hours as described above. At the end of exposure, supernatants were removed, centrifuged and stored at -80°C until use. For analysis, the Human IL-8 Standard ELISA Development Kit from PeproTech (Rocky Hill, USA) was used according to the manufacturer's instructions, downscaled to half of the recommended volumes. Briefly, flat-bottom 96-well plates (Nunc Maxisorp®, eBioscience) were coated with 50 µl IL-8 capture antibody over night (o/n) at room temperature (RT), washed three times and blocked with 1% BSA for 1 hour at RT. After another wash step, 50 µl of a standard series (2000-0 pg/ml) or samples were added in duplicates and left to incubate for 2 hours at RT or o/n at 4°C. Then, the wells were washed again and 50 µl of detection antibody were added for 2 hours at RT. The detection antibody was washed off and 50 µl HRP-streptavidin conjugate (1:2000 in diluent) were added to each well. After incubation for 30 min in

the dark and a final wash step, 50 µl TMB substrate (3,3',5,5'-Tetramethylbenzidine Liquid Substrate, Supersensitive, for ELISA, Sigma-Aldrich) were added to each well and color development was stopped using 50 µl 2M H_2SO_4. Absorbance was recorded at 450 nm with wavelength correction at 650 nm using a microplate reader (Infinite® 200 PRO, Tecan).

Air-liquid interface

Exposure in the ALICE

As described by Lenz *et al.* (2009), the main components of the ALICE are: 1) The exposure chamber in which the cells are exposed, 2) a HEPA-filtered flow system circulates temperature-controlled (37°C), humidified (80-95%) air through the chamber, providing optimal cell culture conditions, 3) the nebulizer, which generates a dense cloud of nanoparticle-containing droplets and 4) the quartz crystal microbalance (QCM), used to monitor deposition of the aerosolized nanoparticles.

Before each exposure, the ZnO nanoparticles were freshly diluted in MilliQ H_2O to concentrations of 0.5 mg/ml and 4.25 mg/ml and supplemented with 500 µM sodium chloride (NaCl) (NAAPREP® physiological saline, GlaxoSmithKline, France) for better aerosol production. A volume of 1 ml of the particle suspension was then nebulized onto the ALI cell cultures placed in the exposure chamber using a vibrating membrane nebulizer (Pari eFlow®*rapid* nebulizer system; PARI GmbH, Starnberg, Germany). At the end of the nebulization process, the cells were left in the exposure chamber until the cloud had settled completely (~15 min), and finally placed in a humidified incubator at 37°C/5% CO_2 for additional incubation periods of 3 or 16 hours at the air-liquid interface.

Cytotoxicity assay

Cytotoxicity was analyzed by measuring the amount of lactate dehydrogenase (LDH) released from damaged cells using the Cytotoxicity Detection Kit (LDH) by Roche (Rotkreuz, Switzerland) according to the distributer's instructions. The medium from the lower compartments was collected and

stored at 4°C until use. For analysis, the samples were centrifuged, a volume of 40 µl of supernatant was transferred into a transparent 96-well flat-bottom plate (Tissue Culture Test Plate 96F, TPP Techno Plastic Products AG) in triplicates and 40 µl Reaction mixture (prepared by mixing 1 part catalyst with 45 parts dye solution) was added. Absorbance was measured at 490 nm with 630 nm as reference, using a Benchmark PLUS micro plate spectrophotometer (Bio-Rad Laboratories). Measurement was repeated several times, until all positive controls were above 1 AU (absorbance units).

Confocal laser scanning microscopy

To complement the cytotoxicity data, the cellular morphology was investigated by confocal laser scanning microscopy (CLSM). After exposure, the cells were fixed for 15 min in 3% paraformaldehyde in PBS at room temperature and transferred to 0.1M glycine in PBS for 10 min, or stored at 4 °C. The cells were washed three times with PBS and subsequently treated with 0.2% Triton X-100 in PBS for 15 min to permeabilize the cell membrane. The actin cytoskeleton of A549, pIL8-GFP and pIL8-Luc cells was stained using AlexaFluor 633 phalloidin, while the cytoskeleton of pIL8-RFP cells was stained using AlexaFluor 488 phalloidin (both 1:100 dilution; Molecular Probes, Life Technologies Europe B.V., Zug, Switzerland). The DNA of all cells was stained using 4',6-diamidin-2-phenylindol (DAPI) at 1 µg/ml in 0.3% Triton X-100 in PBS. Afterwards, the membranes were embedded in Glycergel (DAKO Schweiz AG, Baar, Switzerland) and imaging was performed using an inverted Zeiss confocal laser scanning microscope 710 (LSM, Axio Observer.Z1). For each sample, representative images (z-stacks) were recorded at 5 independent fields of view and further analyzed using the 3D reconstruction software IMARIS (Bitplane AG, Zurich, Switzerland). Submerged samples were prepared by adding the particle suspension directly into the medium covering the cells (26.2 µg/insert; growth area 4.2 cm²) and stained accordingly.

IL8 reporter gene assays

To analyze *IL8* promoter activity of the fluorescent reporter cell lines pIL8-RFP A549 and pIL8-GFP A549, the supernatants were removed and fluorescence intensity of the reporter proteins was measured at $530_{Ex}/590_{Em}$ nm for RFP and $485_{Ex}/535_{Em}$ nm for GFP using a TriStar LB 941 plate reader (Berthold Technologies, Bad Wildbach, Germany). As interference control, a membrane only control was used and subtracted as background fluorescence. To analyze *IL8* promoter activity of the luminescent reporter cell line pIL8-Luc A549, the supernatants were removed and the cells were lysed in 500 µl Luc lysis buffer and stored at -20°C until continuation of the procedure. After thawing, the analysis was continued as described for submerged exposure.

IL8 mRNA expression

IL8 expression at the mRNA level was investigated via qRT-PCR. After removing the supernatants for ELISA and LDH assay, the cells were submerged in RNA Protect Cell Reagent (Qiagen) and stored at -80°C. Samples were thawed on ice and centrifuged at 5000 x *g* for 5 min to remove the RNA Protect Cell Reagent. The pellets were then dissolved in 500 µl TRI Reagent® (Sigma-Aldrich) and total RNA isolation, cDNA synthesis and real-time PCR were performed as described in the submerged exposure section.

IL-8 protein release

The medium from the lower compartment was collected, centrifuged at 16.100x*g* for 15 minutes and the supernatants were stored at -80°C until further analysis. The Human CXCL8/IL-8 DuoSet ELISA Kit from R&D Systems (Minneapolis, MN, USA) was used according to the manufacturer's instructions, downscaled to half of the specified volumes.

Absorbance was measured at 450 nm with 570 nm reference, using a Benchmark PLUS micro plate spectrophotometer (Bio-Rad Laboratories).

Ion release control experiments – submerged cell systems only

pIL8-Luc A549 cells were seeded into 96- or 24-well plates and left to form a monolayer as described above. ZnO-NPs at the appropriate concentrations were diluted in cell culture medium and incubated for 24 hours at 37°C/5% CO_2. Afterwards, the samples were centrifuged at 18.000x*g* / 4°C for 15 minutes to remove the remaining ZnO-NPs. The pellets were discarded while the supernatants containing the ions were pre-warmed and added onto the cells at volumes of 100 µl and 1 ml for 96- and 24-well formats, respectively. As control for ion-induced effects, Zn^{2+} from zinc sulfate heptahydrate ($ZnSO_4 \cdot 7H_2O$, Merck KGaA, Darmstadt, Germany) at a concentration corresponding to the amount of Zn^{2+} released upon total dissolution of the highest ZnO-NP concentration was used (74.76 µg/ml $ZnSO_4 \cdot 7H_2O$ (0.26 µmol/ml) corresponding to 21.2 µg/ml ZnO or 17.03 µg/ml Zn^{2+}). After incubation for 3 or 16 hours, cell viability, cytotoxicity and *IL8* promoter activity by luciferase assay were analyzed as described above.

Results

Characterization of particle deposition at the ALI

Deposition of nebulized NPs was monitored using the integrated quartz crystal microbalance (QCM) within the ALICE. The change in oscillation frequency of the quartz crystal was converted to the deposited mass using the Sauerbrey equation [4]. As shown in **Figure S2**, the deposited masses were 0.62±0.04 µg/cm² for the low ZnO concentration (low_{ZnO}) (0.51 mg/ml stock concentration) and 6.23±0.22 µg/cm² for the high ZnO concentration ($high_{ZnO}$) (4.25 mg/ml stock concentration), yielding deposition efficiencies (*i.e.* the ratio of deposited mass on the bottom of the ALICE to the mass filled into the nebulizer reservoir) of 0.48±0.03 (48±3%) (low_{ZnO}) and 0.58±0.02 (58±2%) ($high_{ZnO}$), respectively.

Effects of ZnO-NP exposure on cellular morphology

Changes in cellular morphology (**Figure S3**) could be observed for cells exposed to $high_{ZnO}$ at the ALI for 16 hours, showing nucleic fragmentation and loss of monolayer integrity. RFP were most affected, similar to what was observed in the LDH assay (by fold-increase). Cells exposed under

submerged conditions did not show any significant changes in cellular morphology. As this may seem contradictory to the results of the LDH assay, it has to be noted that while the concentrations used were matching on surface dose level (6.2 µg/cm²), the administered concentrations were lower for the submerged CLSM exposures (13.1 µg/ml; **Table 1**) than for the LDH exposures.

Flow cytometry analysis – submerged only

As an alternative to the fluorometric readout in the plate reader, the fluorescent cells were also analyzed via flow cytometry. No difference between control cells and ZnO-NP-treated cells at the administered doses of 1.2 (low_{ZnO}) and 11.8 µg/ml ($high_{ZnO}$) for both RFP and GFP could be detected at either time point (**Figure S4**). A slight increase could only be observed when the highest tested dose (21.2 µg/ml, the administered high dose used in the 96-well format) was used, for which after 16 hours a shift in GFP-positive cells from about 32% to 47% could be observed, similar to the small increase seen in the plate reader (data not shown). While this might indicate the flow cytometer to be less sensitive, or rather give less false-positive results (*e.g.* dead cells which usually display more autofluorescence are not included in the assessment), it could also be related to the different doses, since the exposures for the plate reader and flow cytometry analyses were conducted in different well formats and therefore different administered doses were used (**Table 1**). Even though the delivered particle doses per growth area should have been the same (6.2 µg/cm²), ICP-MS measurements showed that most of the ZnO-NPs had already been dissolved at this incubation time (**Table S1**), resulting in a much higher Zn^{2+} concentration in the plate reader (96-well format; 12.69 µg/ml) than in the flow cytometry exposures (24-well format; 7.75 µg/ml), which also might have contributed to the observed "lower" sensitivity of the latter.

Overall, RFP displayed a lower percentage of fluorescent cells in the control sample (~2% RFP-positive cells) compared to GFP (~20-30% GFP-positive cells). Furthermore, treatment with TNF-α for 16 hours resulted in a much higher fold increase in fluorescent cells for RFP (~10-fold compared to

medium treated cells) than for GFP (~2.2-fold over medium control), clearly showing an advantage of RFP over GFP in terms of background levels; assuming the GFP cells were not pre-activated.

Ion release control experiments – submerged only

In order to test the effects of the released ions under submerged conditions, Luc cells were treated with medium pre-incubated with ZnO-NPs, which were removed *via* centrifugation prior to administration of the medium to the cells. For none of the pre-incubated media or the $ZnSO_4$ control effects on cell viability and cytotoxicity could be detected (**Figure S5**). On *IL8* promoter level however, a slight, albeit not significant increase was observed for the highest concentration (2.2±1.0-fold), which was similar to the 2.3±1.3-fold increase obtained from exposure to free Zn^{2+} from $ZnSO_4$ that was administered at a concentration corresponding to the total dissolution of the highest ZnO-NP dose (17.03 µg/ml Zn^{2+}). Hence, even though ICP-MS measurements (**Table S1**) indicated that the NPs did not completely dissolve, the released ions were considered to have substantial impact.

Table S1. ZnO-NP dissolution as measured by ICP-MS

ZnO-NPs [μg/ml]	Total Zn [μg/ml]	ICP-MS Zn [μg/ml]		% dissolved	
		3 hrs	16 hrs	3 hrs	16 hrs
**1.1**	0.9	0.8	0.8	90	87
**2.1**	1.7	1.3	1.3	78	79
**11.8**	9.5	7.0	7.8	74	82
**21.2**	17.0	9.4	12.7	55	75

Table S2. Comparative summary of observed cellular responses and determined endpoints

Suspension	Cytotoxicity (LDH)	Cell Morphology	Fluorescence /Luminescence	*IL8* gene (PCR)	IL-8 protein (ELISA)	Positive control TNF-α
**A549**	ZnO high 16h ↑ (*)	No changes	No fluorescence	ZnO high 3h (↑) (ns)	No changes	3 & 16 h ↑ *IL8* gene (PCR) (***)<br>3 & 16 h ↑ IL-8 protein (ELISA) (***)
**GFP**	ZnO high 16h ↑ (***)	No changes	ZnO high 16h ↑ (ns)	ZnO high 3h (↑) (ns)	No changes	3 & 16 h ↑ Fluorescence (*/***)<br>3 & 16 h ↑ *IL8* gene (PCR) (***/**)<br>3 & 16 h ↑ IL-8 protein (ELISA) (***)
**RFP**	ZnO high 16h ↑ (***)	No changes	No changes	ZnO high 3h (↑) (ns)	No changes	16 h ↑ Fluorescence (***)<br>3 & 16 h ↑ *IL8* gene (PCR) (**/***)<br>3 & 16 h ↑ IL-8 protein (ELISA) (***)
**Luc**	ZnO high 16h (↑) (ns)	No changes	ZnO high 16h ↑ (ns)	ZnO high 3h (↑) (ns)	No changes	3 & 16 h ↑↑ Luminescence (***)<br>3 & 16 h ↑ *IL8* gene (PCR) (***)<br>3 & 16 h ↑ IL-8 protein (ELISA) (***)

ALI	Cytotoxicity (LDH)	Cell Morphology	Fluorescence /Luminescence	*IL8* gene (PCR)	IL-8 protein (ELISA)	Positive control TNF-α
**A549**	ZnO low/high 3h (↑) (ns)<br>ZnO low/high 16h (↑) (ns)/↑ (*)	No changes	No fluorescence	ZnO high 3 & 16h (↑) (ns)	ZnO high 3h (↑) (ns)<br>ZnO high 16h ↑ (**)	3 & 16 h ↑ *IL8* gene (PCR) (**/*)<br>3 & 16 h ↑ IL-8 protein (ELISA) (**/***)
**GFP**	ZnO low/high 3h (↑) (ns)<br>ZnO low/high 16h (↑) (ns)/↑ (*)	No changes	No changes	ZnO high 3 & 16h ↑ (ns/*)	ZnO high 3 & 16h (↑) (ns)	16 h ↑ Fluorescence (ns)<br>3 & 16 h ↑ *IL8* gene (PCR) (ns/*)<br>3 & 16 h ↑ IL-8 protein (ELISA) (***/**)
**RFP**	ZnO low/high 3h (↑) (ns)<br>ZnO low/high 16h (↑) (ns)	ZnO high 16h alterations in morphology	No changes	ZnO high 16h (↑) (ns)	ZnO high 16h (↑) (ns)	16 h ↑ Fluorescence (ns)<br>3 & 16 h ↑ *IL8* gene (PCR) (ns/*)<br>3 & 16 h ↑ IL-8 protein (ELISA) (**/*)
**Luc**	ZnO low/high 3h (↑) (ns)<br>ZnO high 16h ↑ (*)	No changes	No changes	ZnO high 3h ↑ (**)<br>ZnO high 16h (↑) (ns)	ZnO high 3h (↑) (ns)<br>ZnO high 16h ↑ (*)	3 & 16 h ↑ Luminescence (***)<br>3 & 16 h ↑ *IL8* gene (PCR) (**/ns)<br>3 & 16 h ↑ IL-8 protein (ELISA) (**/***)

Figure S1. Characterization of ZnO-NP suspension by TEM. TEM images of NPs in H_2O revealed a non-spherical morphology and mean sizes of 30-40 nm, which agrees with the distributor's information. Mostly single particles were present, however, some agglomerates were also found. In cell culture medium (CCM; RPMI + 10% FCS), mainly large agglomerates were found (two representative TEM images shown).

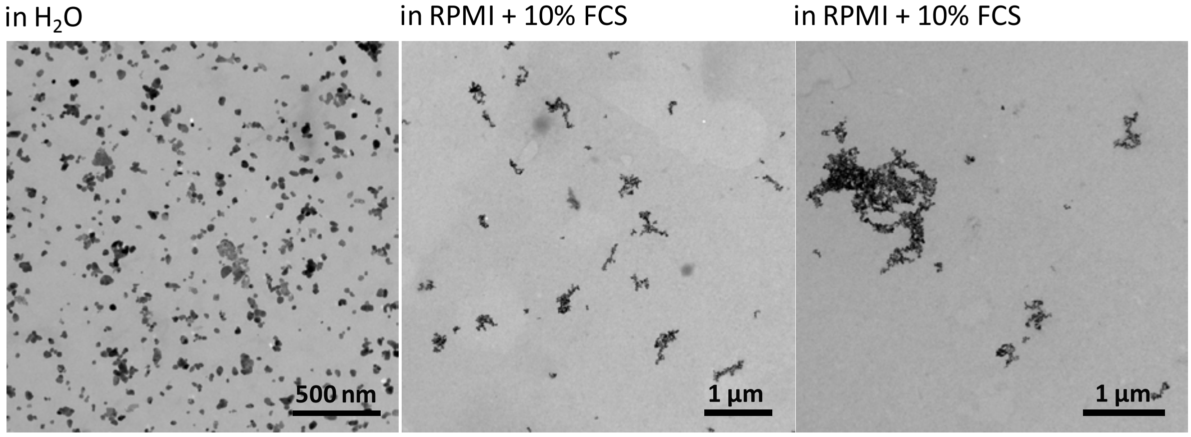

Figure S2. ZnO-NP aerosol deposition. Deposited masses as determined by QCM measurements (A) and deposition efficiencies (B). Data are presented as mean ± SEM as determined from 9 independent experiments. TEM grids placed in the exposure chamber revealed deposition of single particles as well as agglomerates for the lower NP concentration. Scale bars represent 500 nm (C) and 1 µm (D).

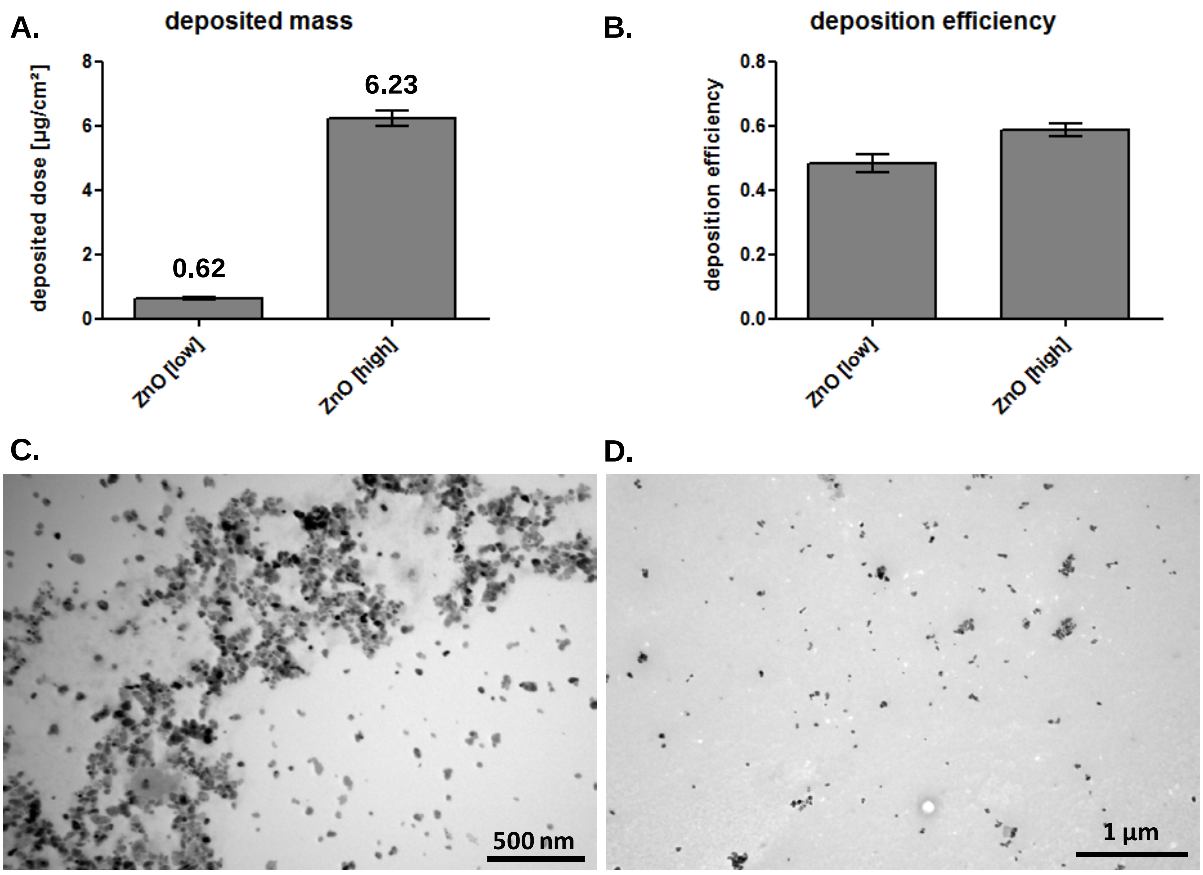

Figure S3. Evaluating cellular morphology under submerged and ALI conditions. CLSM micrographs of cells exposed under submerged and ALI conditions after 16 hrs. The nuclei (labelled with DAPI) are stained blue, the cytoskeleton (F-Actin labelled with phalloidin rhodamine) is stained red.

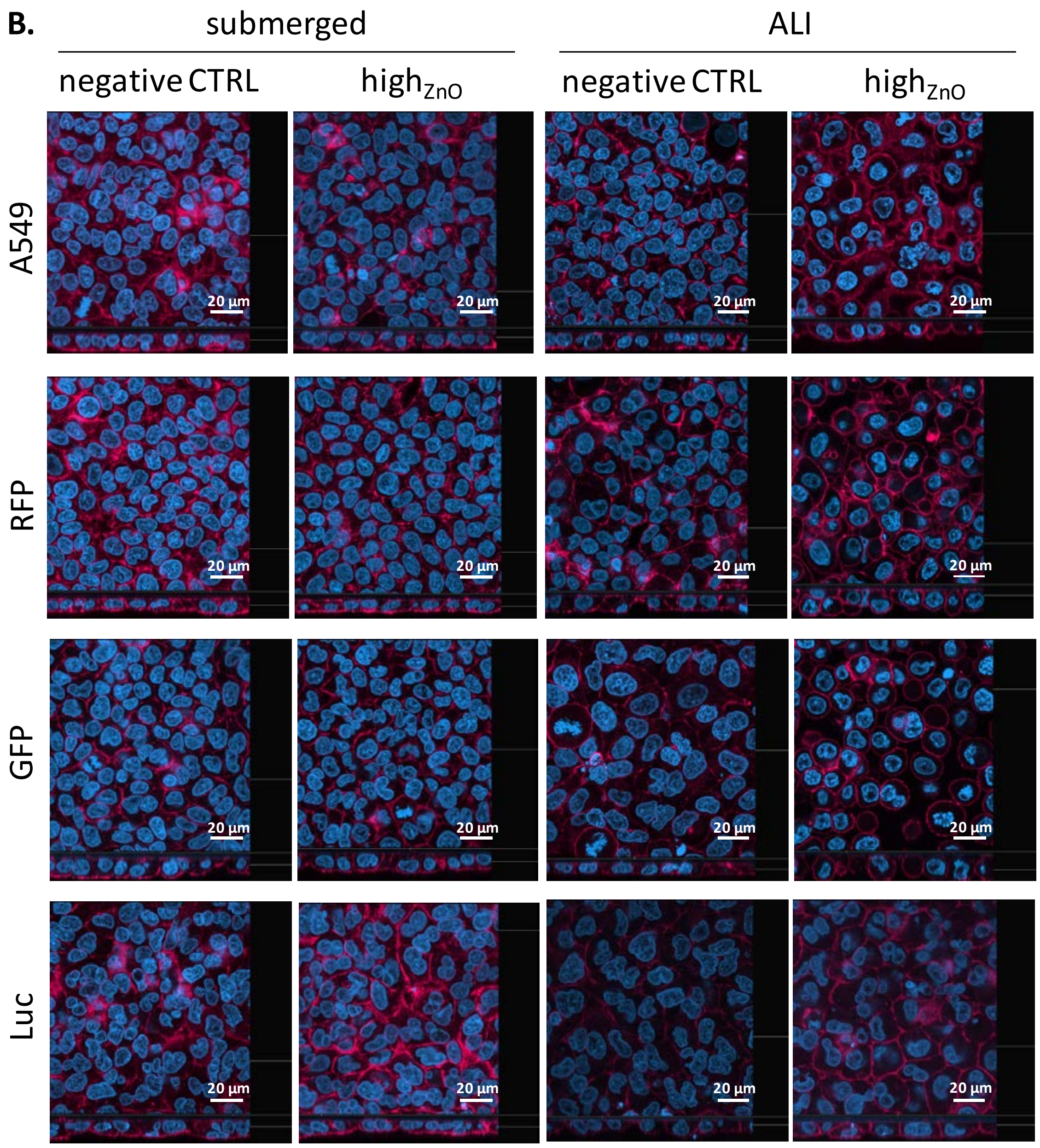

Figure S4. Flow cytometry analysis of pro-inflammatory response upon exposure to ZnO-NPs monitored by fluorescent p*IL8* A549 reporter cell lines under submerged conditions. A. GFP B RFP Data are presented as percentage of GFP-positive and RFP-positive cells, respectively. Error bars indicate the SEM of at least three independent experiments. A one-way analysis of variance (ANOVA) with a subsequent Tukey's Multiple Comparison test was performed. Values were considered significantly different compared to the unexposed (medium) control or as indicated with p<0.05 (*), p<0.001 (**) and p<0.0001 (***).

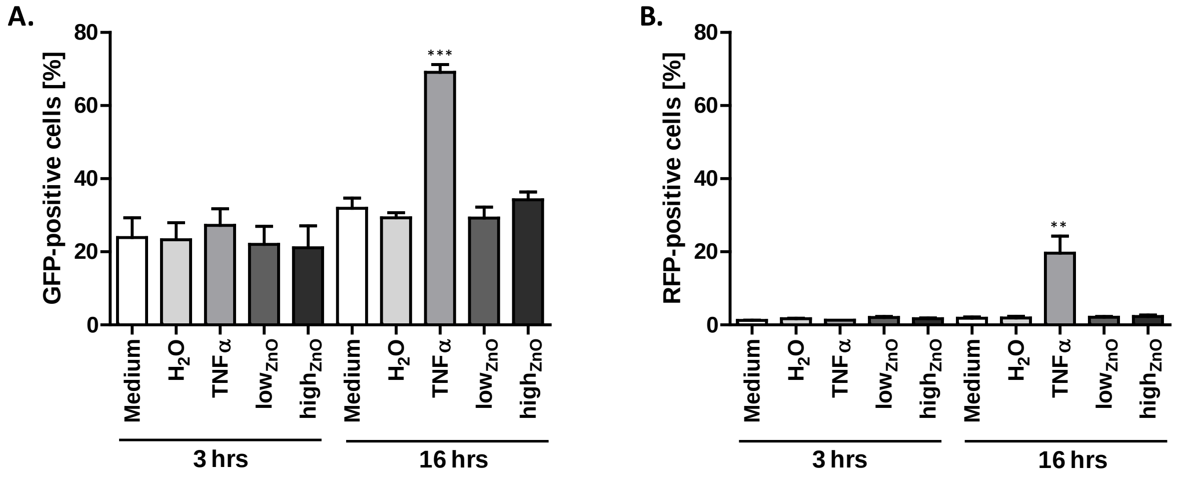

Figure S5. Effects of released Zn^{2+} ions on p*IL8*-Luc A549 cells. Cells were treated with medium pre-incubated with particles for 24 hours at concentrations as indicated or free Zn^{2+} from $ZnSO_4$ (~17 µg/ml) for 3 or 16 hours. A. Cell viability, B. cytotoxicity and C. IL8 promoter activity were analyzed. Data are presented as mean + SEM of two independent experiments performed in triplicates. A one-way analysis of variance (ANOVA) with a subsequent Tukey's Multiple Comparison test was performed. Values were considered significantly different compared to the unexposed control or as indicated with p<0.05 (*), p<0.001 (**) and p<0.0001 (***).

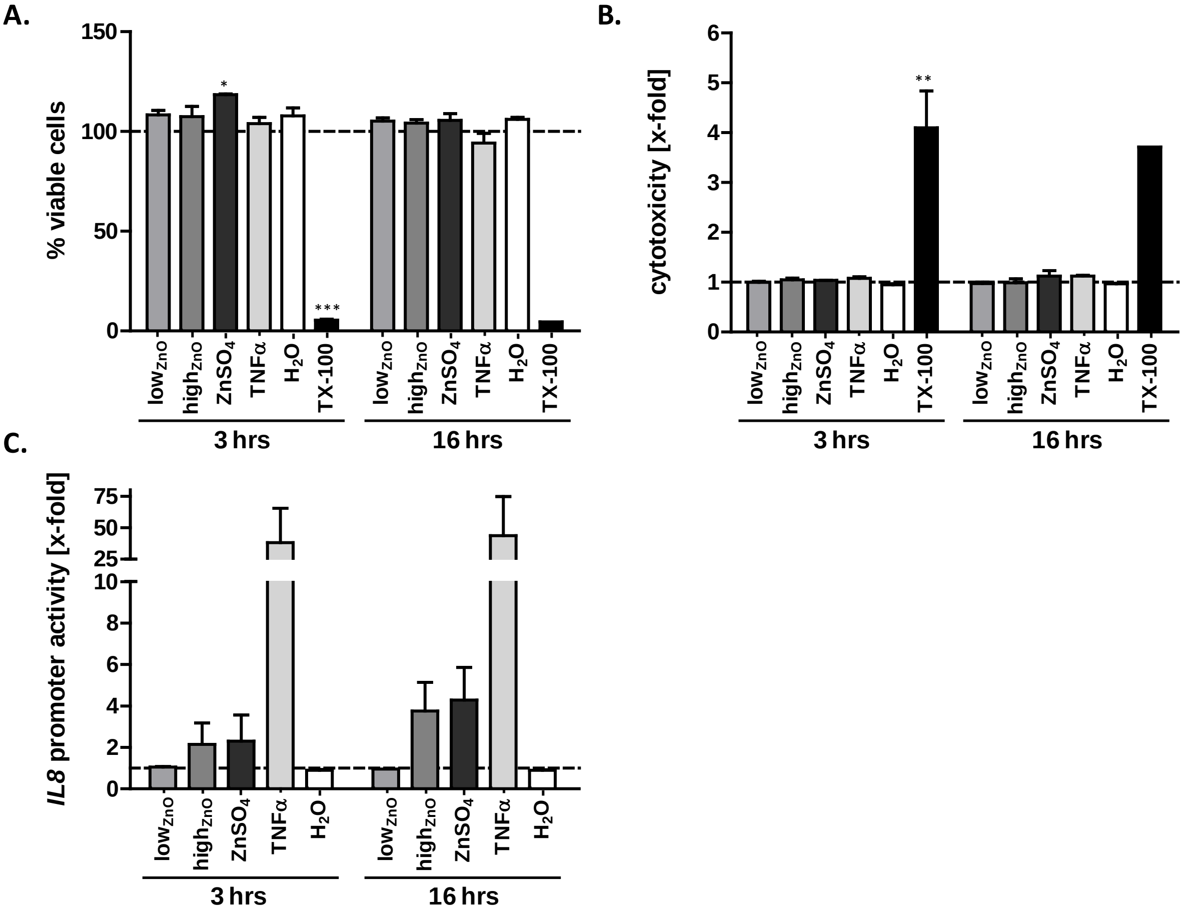

References

1. Lattuada M, Wu H, Morbidelli M. Hydrodynamic radius of fractal clusters. Journal of colloid and interface science. 2003;268(1):96-105.

2. Rodriguez-Lorenzo L, Rothen-Rutishauser B, Petri-Fink A, Balog S. Nanoparticle Polydispersity Can Strongly Affect In Vitro Dose. Particle & Particle Systems Characterization. 2014. doi:10.1002/ppsc.201400079.

3. Oostingh GJ, Schmittner M, Ehart AK, Tischler U, Duschl A. A high-throughput screening method based on stably transformed human cells was used to determine the immunotoxic effects of fluoranthene and other PAHs. Toxicol In Vitro. 2008;22(5):1301-10. doi:S0887-2333(08)00076-3 [pii]

10.1016/j.tiv.2008.03.003.

4. Lenz AG, Karg E, Lentner B, Dittrich V, Brandenberger C, Rothen-Rutishauser B et al. A dose-controlled system for air-liquid interface cell exposure and application to zinc oxide nanoparticles. Part Fibre Toxicol. 2009;6:32. doi:1743-8977-6-32 [pii]

10.1186/1743-8977-6-32.
